# Quantitative Analysis of Differential Proteome Expression in Epithelial-to-Mesenchymal Transition of Bladder Epithelial Cells Using SILAC Method

**DOI:** 10.3390/molecules21010084

**Published:** 2016-01-15

**Authors:** Ganglong Yang, Wei Lu, Di Yu, Chengwen Sun, Jia Guo, Zheng Li, Feng Guan

**Affiliations:** 1 The Key Laboratory of Carbohydrate Chemistry & Biotechnology, Ministry of Education, School of Biotechnology, Jiangnan University, Wuxi 214122, China; glyanglife@jiangnan.edu.cn (G.Y.); luyuweifeng@gmail.com (W.L.); guojia@jiangnan.edu.cn (J.G.); 2 Wuxi Medical School, Jiangnan University, Wuxi 214122, China; jndxyd1988@163.com; 3 Department of Urology, Affiliated Hospital of Jiangnan University, Wuxi 214062, China; chengwensun@163.com; 4 Laboratory for Functional Glycomics, College of Life Sciences, Northwest University, Xi’an 710069, China; zhengli@nwu.edu.cn

**Keywords:** epithelial-to-mesenchymal transition (EMT), bladder cancer, quantitative proteomics, SILAC, mass spectrometry

## Abstract

Epithelial-to-mesenchymal transition (EMT) is an essential biological process involved in embryonic development, cancer progression, and metastatic diseases. EMT has often been used as a model for elucidating the mechanisms that underlie bladder cancer progression. However, no study to date has addressed the quantitative global variation of proteins in EMT using normal and non-malignant bladder cells. We treated normal bladder epithelial HCV29 cells and low grade nonmuscle invasive bladder cancer KK47 cells with transforming growth factor-beta (TGF-β) to establish an EMT model, and studied non-treated and treated HCV29 and KK47 cells by the stable isotope labeling amino acids in cell culture (SILAC) method. Labeled proteins were analyzed by 2D ultrahigh-resolution liquid chromatography/LTQ Orbitrap mass spectrometry. Among a total of 2994 unique identified and annotated proteins in HCV29 and KK47 cells undergoing EMT, 48 and 56 proteins, respectively, were significantly upregulated, and 106 and 24 proteins were significantly downregulated. Gene ontology (GO) term analysis and pathways analysis indicated that the differentially regulated proteins were involved mainly in enhancement of DNA maintenance and inhibition of cell-cell adhesion. Proteomes were compared for bladder cell EMT *vs.* bladder cancer cells, revealing 16 proteins that displayed similar changes in the two situations. Studies are in progress to further characterize these 16 proteins and their biological functions in EMT.

## 1. Introduction

The process of epithelial-to-mesenchymal transition (EMT) plays an important role in the invasion and metastasis of solid cancer cells. During EMT, epithelial cells undergo a transformation into spindle-shaped mesenchymal cells, acquire malignant properties, and become more migratory and invasive [1,2]. The EMT process is characterized by reduced levels of epithelial cell marker molecules (e.g., E-cadherin) and increased levels of mesenchymal markers (e.g., fibronectin, vimentin) [3]. EMT is strongly associated with invasion and metastasis of bladder cancer (BC), the fifth most common type of human cancer [4]. Overall, >70% of BC patients have nonmuscle-invasive disease, while ~25% present initially with muscle invasion. Patients with the muscle-invasive form have a ~50% risk of distant metastases and a poor prognosis [5]. Recurrence of superficial bladder tumors is a major reason for the worldwide prevalence of BC [6]. Intensive studies have identified numerous signaling pathways involved in the molecular mechanisms that underlie bladder carcinogenesis. Among these pathways, most attention has been focused on the transforming growth factor-beta (TGF-β) signaling pathway.

TGF-β signaling plays a crucial role in EMT. TGF-β induces formation of actin stress fibers and production of extracellular matrix (ECM) proteins, including plasminogen activator inhibitor 1 (PAI1) and fibronectin 1 (FN1) [7,8]. During initiation and early stages of tumor development, TGF-β acts as a tumor suppressor by inhibiting cell proliferation and accelerating apoptosis. During later stages, it acts as a tumor promoter by stimulating tumor cell migration and invasion [9]. BC development and progression are often studied using a TGF-β-induced EMT model.

In the rapidly expanding field of systems biology, an essential technique is accurate, precise, and comprehensive measurement of various system components in differentially perturbed states [10]. In-depth analysis of large numbers of proteins has been facilitated by recent advances in proteomics using mass spectrometry (MS) for identification and quantification. Strategies developed for this purpose include 2-dimensional difference gel electrophoresis (2D-DIGE), isotope-coded affinity tagging (ICAT), the similar isobaric tag for relative and absolute quantitation (iTRAQ), and stable isotope labeling by amino acids in cell culture (SILAC) [11,12,13]. Advantages of SILAC over peptide-based absolute quantitation methods include mixing of samples at the very beginning, and reduced variability among samples. SILAC has been widely applied in quantitative proteomic studies of cell biology and many model organisms (yeast, bacteria, plants, mice), and is considered the “gold standard” for protein quantification [14,15,16]. Arginine (Arg) and lysine (Lys) are the stable isotope-labeled amino acids most frequently used in SILAC-based studies, because trypsin digestion of isolated proteins allows MS with a single labeled amino acid, thereby simplifying analysis and quantification [17].

We previously established a model of TGF-β-induced EMT in normal bladder epithelial HCV29 cells [18], and applied a SILAC method for differential proteomic analysis of HCV29, low grade nonmuscle invasive bladder cancer cell line KK47, and metastatic BC cell line YTS1 [19]. In the present study, cultured HCV29 and KK47 cells were labeled with K0R0 (^12^C_6_^14^N_2_-Lys and ^12^C_6_^14^N_4_-Arg), and cells undergoing TGF-β-induced EMT were labeled with K8R10 (^13^C_6_^15^N_2_-Lys and ^13^C_6_^15^N_4_-Arg). Global proteome levels in the two cell lines under the two conditions were quantitatively analyzed and compared.

## 2. Results and Discussions

### 2.1. SILAC Cell Model for Quantification of Proteome in TGF-β-Induced EMT of Bladder Cells

**Figure 1:**
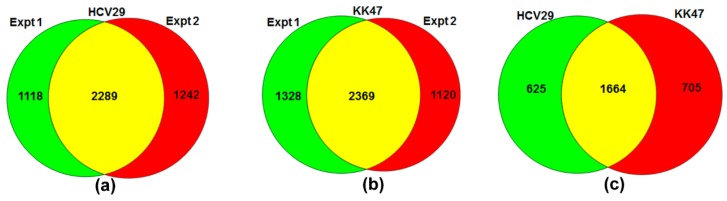
Distributions of proteins identified in experiments described in the text. Venn diagrams of numbers of identified proteins in EMT of HCV29 (**a**) and of KK47 (**b**) from individual experiments, and in EMT of both HCV29 and KK47 (**c**).

Proteins isolated from HCV29 and KK47 cells with and without TGF-β treatment were mixed (in a 1:1 ratio) and digested. Peptides were analyzed by ultra-high-resolution liquid chromatography-tandem MS (nLC-ESI-MS/MS) on a hybrid linear ion trap LTQ Orbitrap. A total of 4649 proteins from HCV29 and 4817 proteins from KK47 were identified in two independent replicate experiments (Figure 1a,b). Of these, 2289 (49.24%) and 2369 (49.18%) proteins that were identified in both experiments and satisfied the criteria for protein quantitation were subjected to further bioinformatic analysis. A total of 1664 proteins were identified in the two cell lines undergoing TGF-β-induced EMT (Figure 1c).

**Figure 2:**
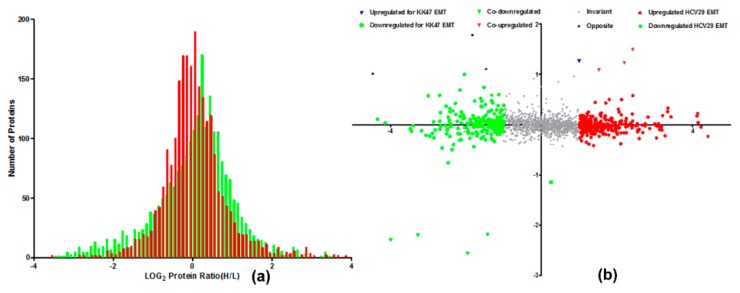
Distribution of SILAC H/L ratios in EMT of HCV29 and KK47 cells. (**a**) Distribution of SILAC H/L ratios; (**b**) Ratios of EMT in HCV29/HCV29 (H/L) and in KK47/KK47 (H/L) for the set of 1664 proteins. log_2_ of the SILAC ratio for each protein (*n* = 2) reflects difference in relative expression in EMT of HCV29 and KK47.

The distribution histograms of H/L log ratios in HCV29 and KK47 cells with and without TGF-β treatment fit a Gaussian distribution. The reproducibility of protein quantitation by SILAC method was characterized and 83.17% of HCV29 and 99.52% of KK47 varied < 2-fold. Therefore, most of the identified proteins were within a ±1 range of log ratios (Figure 2a). Using 1 as the threshold log ratio, expression of three proteins was upregulated and that of four proteins was downregulated during EMT of the two cell lines. Expression of 274 proteins was higher and that of 220 proteins was lower in EMT of HCV29. Expression of one protein was higher and that of one protein was lower in EMT of KK47 (Figure 2b). Interestingly, there were three proteins significantly high-expressed in TGF-β-induced KK47 but significantly low-expressed in TGF-β-induced HCV29, which may be caused by the different response of specific cells to TGF-β. Moreover, the number of dysregulated proteins in EMT of HCV29 was more than that in EMT of KK47. It indicated that non-muscle invasive bladder cancer KK47 cell was more close to carcinoma bladder cancer cells than HCV29.

Population distribution-based z-scores allowed direct comparison of proteins from different experiments. The cutoffs applied were 95%, 99%, and 99.9%, corresponding respectively to z-scores of ±1.960, ±2.576, and ±3.291. With the 95% cutoff, significant differential regulation was observed: 48 upregulated and 106 downregulated proteins in EMT of HCV29, and 56 upregulated and 24 downregulated proteins in EMT of KK47. With the 99% cutoff, we found 16 upregulated and 40 downregulated proteins in EMT of HCV29, and 32 upregulated and 12 downregulated proteins in EMT of KK47. With the 99.9% cutoff, we found five upregulated and five downregulated proteins in EMT of HCV29, 18 upregulated and nine downregulated proteins in EMT of KK47, and three upregulated and four downregulated proteins in EMT of both cell lines (Table 1).

**Table 1:** Protein number, log_2_ ratio, mean ± SD, and z-scores of SILAC-labeled proteins.

Cell Line	log_2_ Mean	log_2_ SD	Z-Scores ^a^		
			±1.960σ	±2.576σ	±3.291σ
HCV29	−0.146	1.298	48, 106	16, 40	5, 5
KK47	−0.005	0.209	56, 24	32, 12	18, 9
Both cell lines			3, 4	3, 4	3, 4

^a^ First and second values: numbers of upregulated and downregulated proteins outside the indicated confidence level.

Table S1 in Supplementary Data I summarizes differentially regulated proteins in EMT of the two cell lines, and their ratios and z-scores.

### 2.2. Functional Classification and Pathway Analysis of Identified Proteins

**Figure 3:**
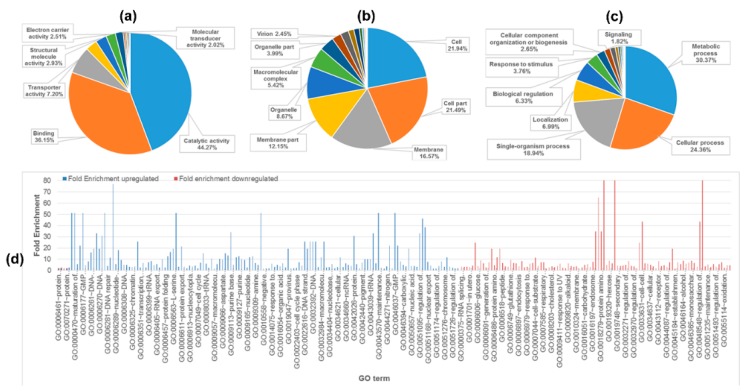
Functional classification and fold enrichment of identified proteins using SWISS-PROT and DAVID databases, based on universal GO annotation terms. Proteins were linked to at least one annotation term within the GO molecular function (**a**); cellular component (**b**); and biological process (**c**) categories; (**d**) Fold enrichment of biological processes in EMT of HCV29 and KK47.

Identified proteins were linked to at least one annotation term each within the GO molecular function, biological process, and molecular component categories, taking into account their nonexclusive localization in GO. The most common molecular functions were catalytic activity (44.27%) and binding (36.15%, including protein, lipid, antigen, and carbohydrate binding) (Figure 3a, Table S2 in Supplementary Data I). The most common cellular component categories were cell (21.94%), cell part (21.49%) (including plasma membrane, cell surface, cell periphery), and membrane (16.57%) (Figure 3b, Table S2 in Supplementary Data I). The most common biological process categories were metabolic process (30.37%), cellular process (24.36%), and single-organism process (13.8%) (Figure 3c, Table S2 in Supplementary Data I).

To identify enrichment terms associated with the upregulated and downregulated groups of proteins, lists of proteins were uploaded to the DAVID website using complete human proteome as background. To determine which biological processes were most affected during EMT, over-represented GO terms were identified based on threshold count ≥2, Expression Analysis Systematic Explorer (EASE) score < 0.1, and *p*-value < 0.05. Fold enrichment in combination with EASE score allowed more comprehensive ranking of the enrichment terms. All fold enrichment values were >2.5. Enrichment biological processes of upregulated proteins in EMT of the two cell lines were associated with base-excision repair, GMP biosynthetic process, lagging strand elongation, and maintenance of DNA methylation. In contrast, enrichment biological processes of downregulated proteins in EMT of the two cell lines were associated with positive regulation of pinocytosis, 4-hydroxyproline metabolic process, cell-cell adhesion mediated by integrin, and protein amino acid N-linked glycosylation via asparagine (Figure 3d). The findings, taken together, indicate that DNA maintenance was enhanced in TGF-β-induced EMT, whereas cell-cell adhesion and pinocytosis were suppressed.

**Figure 4:**
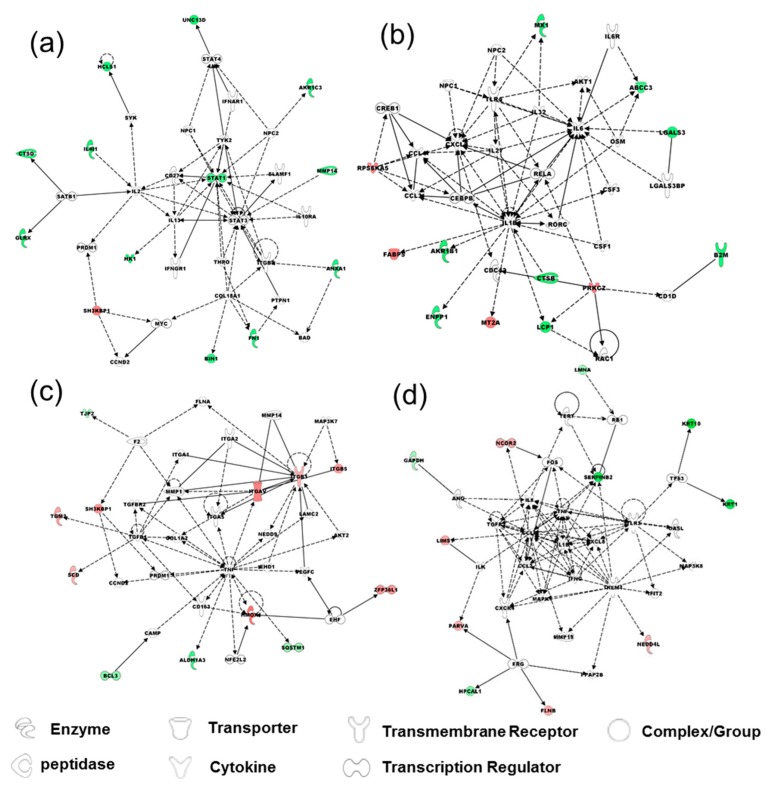
Functional network analysis of differentially regulated proteins with z-score cutoff 95% observed in EMT of HCV29 and KK47 cells, using Ingenuity Pathways Analysis (IPA). Top network functions of (**a**) cellular development, cellular growth and proliferation, hematological system development and function; (**b**) cell death and survival, cell morphology, cellular development in TGF-β-induced EMT of HCV29. Top network functions of (**c**) cancer, cellular movement, tumor morphology; (**d**) cell-to-cell signaling and interaction, tissue development, cell death and survival. Solid lines: direct known interactions. Dashed lines: suspected or indirect interactions. White: proteins known to be in the network but not identified in our study. Red: proteins upregulated in the EMT process. Green: proteins downregulated in the EMT process.

Metabolic and canonical pathways and interconnecting proteins were generated by Ingenuity Pathways Analysis (IPA) following further protein analysis. The results were shown in Table S3 and Table S4 in Supplementary Data I. In brief, the top network functions in EMT of HCV29 cells were cellular development, cellular growth and proliferation, hematological system development and function with one significant upregulated protein (SH3KBP1) and 12 significant downregulated proteins (BIN1, FN1, ANXA1, HK1, GLRX, CTSD, IL4I1, HCLS1, UNC13D, AKR1C3, MMP14, STAT1) (Figure 4a), cell death and survival, cell morphology, cellular development with four significant upregulated proteins (PRKCZ, MT2A, FABP5, FPS6KA5) and eight significant downregulated proteins (ENPP1, LCP1, CTSB, AKR1B1, B2M, LGALS3, ABCC3, MX1) (Figure 4b). The top network functions in EMT of KK47 cells were cancer, cellular movement, tumor morphology with eight significant upregulated proteins (ITGAV, ITGB1, ITGB5, HMOX, SH3KBP1, TGM2, SCD, ZFP36L1) and four significant downregulated proteins (BCL3, ALDH1A3, SQSTM1, TJP2) (Figure 4c), cell-to-cell signaling and interaction, tissue development, cell death and survival with five significant upregulated proteins (LIMS, PARVA, FLNB, NEDD4L, NCOR2) and six significant downregulated proteins (SERPINB2, LMNA, KRT10, KRT1, HPCAL1, GAPDH) (Figure 4d).

### 2.3. Comparison of Proteomes of TGF-β-Induced EMT in Bladder Cells vs. Bladder Cancer Cells

Proteome levels of HCV29, KK47, and YTS1 cells, which mimic various stages of BC, were compared with those observed in TGF-β-induced EMT of HCV29 and KK47. Sixteen proteins (PADI2, UBS3B, ASSY, EF1A2, 1433S, MTAP, B0S8I7, NPM3, PLEK2, 1C07, CATB, ALDR, CKAP4, DPYL3, BIN1, FHL2) were differentially regulated in EMT and in BC cells. ASSY (argininosuccinate synthase) was upregulated in EMT of HCV29 (log_2_ H:L = 3.34) and KK47 (log_2_ H:L = 0.26), as well as in BC cells (log_2_ KK47:HCV29 = 3.47; log_2_ YTS1:HCV29 = 0.89). ASSY is the key enzyme involved in Arg metabolism, and is also expressed highly in colorectal, gastric, and lung squamous cell cancers [20]. Elongation factor 1 alpha-2 (EF1A2), a protein translation factor involved in protein synthesis, tumorigenesis, and tumor progression, is upregulated in ovarian, breast, gastric, and non-small cell lung cancers [21,22]. EF1A2 was overexpressed in both EMT process (log_2_ H/L ratios 3.20 for HCV29, 0.18 for KK47) and BC cells (log_2_ ratios 4.14 for KK47/HCV29, −0.20 for YTS1/HCV29). Other proteins upregulated in both ETM and BC (indicated in Table S1 in Supplementary Data I by *) included SFN (14-3-3 protein sigma) and PLEK2 (pleckstrin-2).

**Figure 5:**
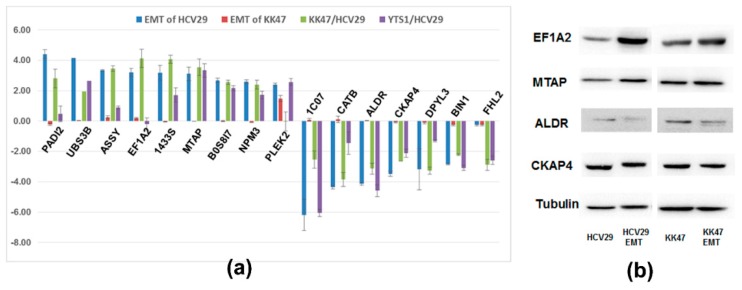
Comparison of proteomes of TGF-β-induced EMT in bladder cells *vs.* bladder cancer cells. (**a**) The SILAC ratio of proteins in TGF-β-induced EMT bladder cells *vs.* bladder cancer cells. (**b**) Western blot analysis. PADI2: Protein-arginine deiminase type-2; UBS3B: Ubiquitin-associated and SH3 domain-containing protein B; ASSY: Argininosuccinate synthase; EF1A2: Elongation factor 1-alpha 2; 1433S: 14-3-3 protein sigma; MTAP: S-methyl-5-thioadenosine phosphorylase; B0S8I7: L antigen family member 3; NPM3: Nucleoplasmin-3; PLEK2: Pleckstrin-2; 1C07: HLA class I histocompatibility antigen; CATB: Cathepsin B; ALDR: Aldose reductase; CKAP4: Cytoskeleton-associated protein 4; DPYL3: Dihydropyrimidinase-related protein 3; BIN1: Myc box-dependent-interacting protein 1; FHL2: Four and a half LIM domains protein 2.

On the other hand, CKAP4 (cytoskeleton-associated protein 4) was downregulated in EMT of HCV29 (log_2_ H:L = −3.47) and KK47 (log_2_ H:L = −0.08), and also in BC cells (log_2_ KK47:HCV29 = −2.64, log_2_ YTS1:HCV29 = −2.12). CKAP4 mediates anchoring of the endoplasmic reticulum to microtubules and is a high-affinity epithelial cell surface receptor for anti-proliferative factor. Downregulation of CKAP4 accelerates the EMT process and cancer cell proliferation. HLA-C (HLA class I histocompatibility antigen) plays crucial roles in immune recognition of transformed and virus-infected cells. It binds to “non-self” or aberrantly expressed proteins, presents the newly formed complex to T lymphocytes, and initiates a series of immune reactions leading to elimination of tumor cells by cytotoxic T cells [23]. Downregulation of HLA-C was observed in patients with non-small cell lung cancer [24]. In the present study, HLA-C was downregulated in EMT of HCV29 (log_2_ H:L = −6.19) and KK47 (log_2_ H:L = 0.07), as well as in BC cells (log_2_ KK47:HCV29 = −2.53, log_2_ YTS1:HCV29 = −6.05). Other proteins downregulated in both ETM and BC (indicated in Table S1 Supplementary Data I by #) included CTSB (cathepsin B) and AKR1B1 (aldose reductase) (Figure 5a).

Four proteins in the 16 proteins were selected and confirmed by western blot. EF1A2 and MTAP proteins were detected at higher levels in TGF-β-induced EMT of HCV29 than in HCV29 cell, whereas ALDR and CKAP4 proteins were detected at lower levels in TGF-β-induced EMT cells than in HCV29 cell. At the same time, the expression of EF1A2, MTAP, ALDR and CKAP4 proteins had no significant difference between the TGF-β-induced EMT of KK47 and KK47 cell (Figure 5b). In general, the western blot results were consistent with the variables from MS analysis.

## 3. Experimental Section

### 3.1. Cell Culture

HCV29 and KK47 cells were kindly donated by Dr. Sen-itiroh Hakomori (The Biomembrane Institute, Seattle, WA, USA). Cells were cultured in RPMI 1640 medium supplemented with 10% FBS and 1% penicillin/streptomycin at 37 °C in 5% CO_2_ atmosphere. For SILAC labeling, cells were cultured in SILAC-labeled RPMI 1640 with 10% FBS and 1% penicillin/ streptomycin containing “light” (K0R0) or “heavy” (K8R10) Lys and Arg. L-Pro (200 mg/L) was added to the medium to prevent Arg-to-Pro conversion [25]. Cells were cultured for at least five passages to eliminate nonlabeled Lys and Arg. “Heavy” labeled cells were seeded in “heavy” culture medium overnight until ~30% confluence, and then stimulated with 2 ng/mL TGF-β1 (BD Biosciences; San Jose, CA, USA) for 48 h.

### 3.2. Cell Lysis and Protein Extraction

Total proteins of labeled cells were lysed and extracted using Tissue Protein Extraction Reagent (T-PER) (Thermo Scientific; San Jose, CA, USA). In brief, cells (~1 × 10^7^) were detached with trypsin, washed twice with ice-cold 1 × PBS (0.01 M phosphate buffer containing 0.15 M NaCl, pH 7.4), lysed with 1 mL T-PER containing protease inhibitors (1 mM PMSF, 0.1% aprotinin), incubated for 30 min on ice, homogenized, and centrifuged at 12,000 rpm for 15 min. The supernatant was stored at −80 °C. Protein concentration was determined by BCA assay (Beyotime; Haimen, China).

### 3.3. In-Solution Digestion

Stable isotope-labeled proteins from TGF-β-treated and -untreated cells were mixed at 1:1, reduced, and alkylated by incubation with 10 mM dithiothreitol (DTT) and 20 mM iodoacetamide (IAM). Alkylated proteins were digested by trypsin at ratio 1:50 (*w*/*w*) and incubated overnight at 37 °C [26]. Total peptides were concentrated and desalted using a 10 KD size-exclusion spin ultrafiltration unit and dried using a SpeedVac concentrator.

### 3.4. LC-MS/MS Analysis

2D-LC-MS was performed using an LTQ Orbitrap mass spectrometer (Thermo Fisher Scientific; Waltham, MA, USA) as described previously [19,27]. Digested peptides (100 µg) were injected into a biphasic capillary column (i.d. 200 µm) packed with C_18_ resin (ReproSil-Pur, 5 µm, Maisch GmbH) and strong cation-exchange resin (Luna 5 µm SCX 100A, Torrance, CA, USA). Peptide effluents were direct-piped into a 15-cm C_18_ analytical column (i.d. 75 µm, ReproSil-Pur, 3 µm) at flow rate 500 nL/min. Nano-ESI MS was performed at spray voltage 2.0 kV and heated capillary temperature 200 °C. One full MS scan (300–1800) in the Orbitrap was followed by MS/MS scans of the five most intense ions selected from the MS spectrum in LTQ. Charge state screening was assigned for +2, +3, +4, and above [28].

### 3.5. Data Analysis

Raw MS data were analyzed using the MaxQuant software program (V. 1.2.2.5) [29,30]. False discovery rate (FDR) 0.01 for proteins and peptides and minimum peptide length 6 amino acids were required. MS/MS spectra were annotated by the Andromeda search engine [31] against the International Protein Index (IPI) human database (V. 3.85). SILAC state of peptides was determined by MaxQuant from mass differences between SILAC peptide pairs, and these data were used to perform searches with fixed Arg10 and Lys8 modifications, as appropriate. Quantification in MaxQuant was performed as described previously [29].

Differential regulation within each experimental and H/L (“heavy/ light”) ratio of the identified proteins was normalized using z-score analysis, as described previously [32,33]. The log_2_ H/L ratio of each protein was converted into a z-score, using the formula:

$$z-\text{score}\left(\sigma \right)\;\text{of}\;\left[b\right]=\frac{\log_{2}\frac{X\left(H\right)}{L}\left[b\right]-Average\;of\;\left(\log_{2}\;of\;each\;number,\;a\ldots n\right)}{Standard\;deviation\;of\;\left(\log_{2}\;of\;each\;number,\;a\ldots n\right)}$$

where *b* represents a single protein in a data set population (*a*…*n*). A z-score ≥1.960σ indicates that differential expression of the protein lies outside the 95% confidence interval, a score ≥2.576σ indicates expression outside the 99% confidence interval, and a score ≥3.291σ means 99.9% confidence. Z-scores ≥1.960σ were considered to be significant.

### 3.6. Functional Annotation and Ingenuity Pathways Analysis

Identified proteins were analyzed using the SWISS-PROT database for classification according to biological process, cellular component, and molecular function [34]. Significant over-represented gene ontology (GO) terms were annotated using the Database for Annotation, Visualization, and Integrated Discovery (DAVID) gene bioinformatic resources [35,36]. Each protein IPI number was mapped to its corresponding gene object in the Ingenuity Pathways Knowledge Base (Ingenuity Systems) [37]. Networks among the proteins were generated algorithmically based on connectivity.

### 3.7. Differential Analysis of Proteomes of TGF-β-Induced EMT in Bladder Cells vs. Bladder Cancer Cells and Validation by Western Blot

To reveal relevance of the TGF-β-induced EMT with bladder cancer, the proteome levels in TGF-β-induced HCV29 and KK47 cells were compared with HCV29, KK47, and YTS1 cells, which were previous used to elucidate the protein alteration in the bladder cancer [19]. 16 proteins in TGF-β-induced EMT expressed accordance with bladder cancer cells. Then 4 proteins were validated by western blot, which include the antibody of EF1A2, MTAP, ALDR, and CKAP4 (ABclonal Technology, Wuhan, China).

## 4. Conclusions

We successfully applied the SILAC method for identification and quantification of aberrantly regulated proteins during EMT of two bladder cell lines. Studies are in progress to elucidate the functional roles and mechanisms of these proteins in bladder cancer.

## Supplementary Materials

Supplementary materials can be accessed at: http://www.mdpi.com/1420-3049/21/1/84/s1.
