# Supplementary material for: Quantitative Analysis of Differential Proteome Expression in Epithelial-to-Mesenchymal Transition of Bladder Epithelial Cells Using SILAC Method
Source: Molecules. 2016 Jan 15;21(1):84. doi: 10.3390/molecules21010084 (PMC6273313; doi:10.3390/molecules21010084)
Supplement: Supplementary file 1 [file molecules-21-00084-s001.zip › molecules-108273/molecules-108273-supplementary-data I.pdf]

Supplementary Data I: Quantitative Analysis of Differential Proteome Expression in Epithelial-to-Mesenchymal Transition of Bladder Epithelial Cells Using SILAC Method

Ganglong Yang [1], Wei Lu [1], Di Yu [2], Chengwen Sun [3], Jia Guo [1], Zheng Li [4] and Feng Guan [1,*]

Table S1. Differential expression of proteins in EMT of HCV29 and KK47 with at least one z-score value (H/L) >1.960 σ in two biological replicates.

Swiss-Prot	Protein Name	Gene Name	EMT of HCV29			EMT of KK47		
			H/L Mean	$\log_2$ Mean	Z-Score	H/L Mean	$\log_2$ Mean	Z-Score
EBP_HUMAN	3-beta-hydroxysteroid-delta(8)	EBP	37.54	5.23	4.14	1.00	0.00	0.02
PADI2_HUMAN *	Protein-arginine deiminase type-2	PADI2	21.42	4.40	3.51	0.85	−0.23	−1.09
CBS_HUMAN	Cystathionine beta-synthase	CBS	33.13	4.22	3.37	0.97	−0.05	−0.20
UBS3B_HUMAN *	Ubiquitin-associated and SH3 domain-containing protein B	UBASH3B	17.63	4.14	3.30	1.03	0.04	0.23
Q5JB52_HUMAN	Heterogeneous nuclear ribonucleoprotein L-like	HNRPLL	17.58	4.14	3.30	1.17	0.23	1.10
1A36_HUMAN	HLA class I histocompatibility antigen	HLA-A	17.34	4.12	3.28	1.13	0.17	0.85
MT2_HUMAN	Metallothionein-2	MT2A	11.15	3.43	2.75	1.11	0.16	0.77
H0Y4M8_HUMAN	Zinc finger protein 185	ZNF185	10.78	3.35	2.69	1.26	0.33	1.60
ASSY_HUMAN *	Argininosuccinate synthase	ASS1	10.13	3.34	2.69	1.20	0.26	1.25
IMUP_HUMAN	Immortalization upregulated protein	IMUP	10.35	3.27	2.63	0.86	−0.22	−1.03
EF1A2_HUMAN *	Elongation factor 1-alpha 2	EEF1A2	9.29	3.20	2.58	1.14	0.18	0.90
RBM27_HUMAN	RNA-binding protein 27	RBM27	9.38	3.18	2.57	0.93	−0.11	−0.50
1433S_HUMAN *	14-3-3 protein sigma	SFN	9.28	3.17	2.56	0.97	−0.04	−0.18
CLMN_HUMAN	Calmin	CLMN	8.73	3.12	2.52	0.93	−0.10	−0.45
MTAP_HUMAN *	S-methyl-5-thioadenosine phosphorylase	MTAP	8.86	3.11	2.51	1.00	−0.01	−0.01
IF2B1_HUMAN	Insulin-like growth factor 2 mRNA-binding protein 1	IGF2BP1	8.83	3.10	2.50	1.01	0.02	0.11
J3KT73_HUMAN	60S ribosomal protein L38	RPL38	12.87	3.09	2.49	0.98	−0.03	−0.14
SPY4_HUMAN	Protein sprouty homolog 4	SPRY4	8.52	3.08	2.49	0.98	−0.03	−0.10
KS6A5_HUMAN	Ribosomal protein S6 kinase	RPS6KA5	8.36	3.04	2.46	0.83	−0.26	−1.23
ARRB2_HUMAN	Beta-arrestin-2	ARRB2	8.19	3.03	2.45	0.99	−0.02	−0.07
L2GL2_HUMAN	Lethal(2) giant larvae protein homolog 2	LLGL2	8.10	3.00	2.42	1.02	0.02	0.10
SSRP1_HUMAN	FACT complex subunit SSRP1	SSRP1	7.28	2.86	2.32	0.95	−0.08	−0.33
E9PES6_HUMAN	High mobility group protein B3	HMGB3	7.06	2.82	2.29	1.11	0.15	0.74

Table S1. *Cont.*

Swiss-Prot	Protein Name	Gene Name	EMT of HCV29			EMT of KK47		
			H/L Mean	log_2 Mean	Z-Score	H/L Mean	log_2 Mean	Z-Score
SMHD1_HUMAN	Structural maintenance of chromosomes flexible hinge domain-containing protein 1	SMCHD1	6.98	2.76	2.24	1.00	−0.01	−0.01
G3V5B2_HUMAN	Legumain	LGMN	6.73	2.74	2.23	1.01	0.02	0.10
C9JXC1_HUMAN	Ribonucleoside-diphosphate reductase subunit M2	RRM2	6.97	2.74	2.22	0.99	−0.03	−0.09
NO66_HUMAN	Bifunctional lysine-specific demethylase and histidyl-hydroxylase NO66	NO66	6.61	2.68	2.18	0.90	−0.16	−0.72
B0S8I7_HUMAN *	L antigen family member 3	LAGE3	6.37	2.67	2.17	0.99	−0.01	−0.01
NPM3_HUMAN *	Nucleoplasmin-3	NPM3	6.02	2.59	2.11	0.94	−0.09	−0.41
P20D2_HUMAN	Peptidase M20 domain-containing protein 2	PM20D2	6.09	2.58	2.10	0.83	−0.28	−1.29
TRM61_HUMAN	tRNA (adenine(58)-N(1))-methyltransferase catalytic subunit TRMT61A	TRMT61A	6.06	2.55	2.08	0.93	−0.11	−0.49
TRM6_HUMAN	tRNA (adenine(58)-N(1))-methyltransferase non-catalytic subunit TRM6	TRMT6	5.92	2.55	2.08	0.93	−0.11	−0.49
ITPA_HUMAN	Inosine triphosphate pyrophosphatase	ITPA	5.83	2.54	2.07	0.91	−0.14	−0.65
SYDM_HUMAN	Aspartate-tRNA ligase, mitochondrial	DARS2	6.02	2.54	2.07	0.99	−0.01	−0.04
DCTP1_HUMAN	dCTP pyrophosphatase 1	DCTPP1	5.82	2.54	2.07	1.01	0.02	0.12
H7BYU9_HUMAN	UPF0687 protein C20orf27	C20orf27	5.66	2.49	2.03	0.99	−0.02	−0.06
PYRG1_HUMAN	CTP synthase 1	CTPS1	5.35	2.42	1.97	1.49	0.58	2.80
F5GZ28_HUMAN	DNA ligase; DNA ligase 1	LIG1	5.35	2.41	1.97	1.05	0.07	0.38
DIAP3_HUMAN	Protein diaphanous homolog 3	DIAPH3	5.30	2.40	1.96	1.16	0.21	1.03
1C07_HUMAN #	HLA class I histocompatibility antigen	HLA-C	0.02	−6.19	−4.66	1.06	0.07	0.38
CATB_HUMAN #	Cathepsin B	CTSB	0.05	−4.34	−3.23	1.09	0.11	0.53
ALDR_HUMAN #	Aldose reductase	AKR1B1	0.06	−4.13	−3.07	1.02	0.03	0.16
CKAP4_HUMAN #	Cytoskeleton-associated protein 4	CKAP4	0.09	−3.47	−2.56	0.95	−0.08	−0.36
MGLL_HUMAN	Monoglyceride lipase	MGLL	0.09	−3.44	−2.54	0.91	−0.13	−0.60
SCRB2_HUMAN	Lysosome membrane protein 2	SCARB2	0.10	−3.40	−2.51	1.00	−0.02	−0.07
DPYL3_HUMAN #	Dihydropyrimidinase-related protein 3	DPYSL3	0.14	−3.17	−2.33	0.92	−0.12	−0.56
ANXA1_HUMAN	Annexin A1	ANXA1	0.12	−3.06	−2.24	0.86	−0.21	−0.98
A8MWK3_HUMAN	Cadherin-2	CDH2	0.13	−3.00	−2.20	1.49	0.57	2.77
MYO1B_HUMAN	Unconventional myosin-Ib	MYO1B	0.13	−2.96	−2.17	0.86	−0.23	−1.06
TBD2A_HUMAN	TBC1 domain family member 2A	TBC1D2	0.17	−2.89	−2.12	0.89	−0.18	−0.81
SERPH_HUMAN	Serpin H1	SERPINH1	0.14	−2.86	−2.09	1.04	0.06	0.31
BIN1_HUMAN #	Myc box-dependent-interacting protein 1	BIN1	0.14	−2.85	−2.08	0.83	−0.26	−1.23
VIME_HUMAN	Vimentin	VIM	0.14	−2.82	−2.06	0.94	−0.11	−0.49
S10A6_HUMAN	Protein S100-A6	S100A6	0.15	−2.70	−1.97	1.51	0.58	2.82
STAT1_HUMAN	Signal transducer and activator of transcription 1-alpha/beta	STAT1	0.16	−2.70	−1.96	1.05	0.07	0.37
NMES1_HUMAN	Normal mucosa of esophagus-specific gene 1 protein	NMES1	2.02	1.00	0.88	2.42	1.27	6.09
EPHA2_HUMAN	Ephrin type-A receptor 2	EPHA2	1.20	0.25	0.31	0.45	−1.14	−5.43
H7C4E5_HUMAN	Cytochrome c oxidase copper chaperone	COX17	2.91	1.52	1.28	2.16	1.09	5.24
PLEK2_HUMAN *	Pleckstrin-2	PLEK2	5.32	2.41	1.97	2.81	1.49	7.15

Table S1. *Cont.*

Swiss-Prot	Protein Name	Gene Name	EMT of HCV29			EMT of KK47		
			H/L Mean	log_2 Mean	Z-Score	H/L Mean	log_2 Mean	Z-Score
Q5QPE4_HUMAN	Sorting nexin-5	SNX5	4.59	2.20	1.81	2.45	1.23	5.92
K1C10_HUMAN	Keratin, type I cytoskeletal 10	KRT10	0.26	−1.96	−1.40	0.17	−2.56	−12.24
K2C1_HUMAN	Keratin, type II cytoskeletal 1	KRT1	0.52	−1.43	−0.99	0.24	−2.19	−10.45
K7EQQ3_HUMAN	Keratin, type I cytoskeletal 9	KRT9	0.07	−3.99	−2.96	0.21	−2.29	−10.93
PAI2_HUMAN	Plasminogen activator inhibitor 2	SERPINB2	0.11	−3.27	−2.41	0.23	−2.20	−10.49
FHL2_HUMAN [#]	Four and a half LIM domains protein 2	FHL2	0.05	−4.46	−3.33	2.02	1.02	4.88
HM13_HUMAN	Minor histocompatibility antigen H13	HM13	0.29	−1.83	−1.30	3.57	1.79	8.57
ITAV_HUMAN	Integrin alpha-V	ITGAV	0.39	−1.46	−1.02	2.16	1.10	5.29

* Proteins showing upregulated expression in BC cells and EMT of HCV29/KK47; [#] Proteins showing downregulated expression in BC cells and EMT of HCV29/KK47.

Table S2. The GO data of the proteins in EMT of HCV29 and KK47.

Molecular_Function	25408783	Percentage	Cellular_Component	13892518	Percentage	Biological_Process	26697308	Percentage
Catalytic activity	17325358	44.27%	Cell	9444003	21.94%	Metabolic process	21019512	30.37%
Binding	14147991	36.15%	Cell part	9248594	21.49%	Cellular process	16858784	24.36%
Transporter activity	2818072	7.20%	Membrane	7132261	16.57%	Single-organism process	13106784	18.94%
Nucleic acid binding transcription factor activity	1192117	3.05%	Membrane part	5228028	12.15%	Localization	4840884	6.99%
Structural molecule activity	1145892	2.93%	Organelle	3732192	8.67%	Biological regulation	4382130	6.33%
Electron carrier activity	981781	2.51%	Macromolecular complex	2332064	5.42%	Response to stimulus	2603498	3.76%
Molecular transducer activity	792386	2.02%	Organelle part	1717532	3.99%	Cellular component organization or biogenesis	1835449	2.65%
Molecular function regulator	191975	0.49%	Virion	1056179	2.45%	Signaling	1256404	1.82%
Protein binding transcription factor activity	163669	0.42%	Virion part	951609	2.21%	Multi-organism process	1145260	1.65%
Antioxidant activity	160684	0.41%	Other organism	637466	1.48%	Locomotion	618986	0.89%
Enzyme regulator activity	146128	0.37%	Other organism part	637465	1.48%	Biological adhesion	453915	0.66%
Guanyl-nucleotide exchange factor activity	38653	0.10%	Extracellular region	329430	0.77%	Multicellular organismal process	356047	0.51%
Nutrient reservoir activity	10769	0.03%	Membrane-enclosed lumen	242080	0.56%	Developmental process	340209	0.49%
Channel regulator activity	8873	0.02%	Extracellular region part	160174	0.37%	Immune system process	182261	0.26%
Translation regulator activity	1894	0.00%	Cell junction	53256	0.12%	Reproduction	84740	0.12%
D-alanyl carrier activity	1260	0.00%	Extracellular matrix	41673	0.10%	Reproductive process	65167	0.09%
Metallochaperone activity	1256	0.00%	Synapse	28447	0.07%	Growth	27656	0.04%
Protein tag	1143	0.00%	Nucleoid	25684	0.06%	Behavior	19580	0.03%
Receptor regulator activity	1066	0.00%	Synapse part	20728	0.05%	Rhythmic process	9606	0.01%
Chemoattractant activity	709	0.00%	Collagen trimer	10304	0.02%	Cell killing	5309	0.01%
Chemorepellent activity	306	0.00%	Plasmodesma	6066	0.01%	Hormone secretion	2388	0.00%
Morphogen activity	123	0.00%	Extracellular matrix component	4166	0.01%	Cell aggregation	1086	0.00%
			Viral occlusion body	495	0.00%	Biological phase	76	0.00%

Table S3. The IPA data in the EMT of HCV29 cells.

ID	Molecules in Network	Score	Focus Molecules	Top Diseases and Functions
1	AKR1C3,ANXA1,BAD,BIN1,CCND2,CD274,COL18A1,CTSD,FN1,GLRX,HCLS1,HK1, IFNAR1,IFNGR1,IL2,IL13,IL10RA,IL4I1,ITGB2,MMP14,MYC,NPC1,NPC2,PRDM1, PTPN1,SATB1,SH3KBP1,SLAMF1,STAT1,STAT3,STAT4,SYK,THPO,TYK2,UNC13D	14	3	Cellular Development, Cellular Growth and Proliferation, Hematological System Development and Function
2	AKT1,ARRB2,ATP2B1,CCL5,CD4,CD28,CD40,CSF2,CXCL10,GBP1,HLA-B,HLA-C, IFNA1/IFNA13,IFNAR2,IFNG,IL6,IL10,IL18,IL1B,ISG15,KIR3DL1,MME,NPM3,OAS2, OSM,PLG,PSMB10,PSME2,RRM2,SERPINB2,TLR3,TLR4,TNF,TREM1,UBA7	12	3	Dermatological Diseases and Conditions, Inflammatory Response, Cell-To-Cell Signaling and Interaction
3	ABCC3,AKR1B1,AKT1,B2M,CCL3,CCL4,CD1D,CDC42,CEBPB,CREB1,CSF1,CSF3, CTSB,CXCL8,ENPP1,FABP5,IL6,IL27,IL32,IL1B,IL6R,LCP1,LGALS3,LGALS3BP, MT2A,MX1,NPC1,NPC2,OSM,PRKCZ,RAC1,RELA,RORC,RPS6KA5,TLR4	12	2	Cell Death and Survival, Cell Morphology, Cellular Development
4	AR,CAPG,CAV1,CCL2,CCL5,CDKN1A,CDKN2A,CKAP4,COL6A3,CXCR4,DCTPP1, DIAPH3,ERG,FBN1,IFNG,IL6,IL1B,ITPA,MMP1,MMP3,MMP9,MMP25,MYL9,NOS3, RELA,SERPINH1,SMAD3,SP1,SPARC,SRF,TERT,TGFB1,TNF,TP53,VIM	11	3	Cellular Development, Cellular Growth and Proliferation, Skeletal and Muscular System Development and Function
5	CASP14,OCIAD2	1	0	Cell-To-Cell Signaling and Interaction, Tissue Development, Cell Morphology
6	LIPE,PTRF	1	1	Carbohydrate Metabolism, Molecular Transport, Small Molecule Biochemistry
7	HIF1A,PLOD2	1	1	Organismal Injury and Abnormalities, Connective Tissue Disorders, Hereditary Disorder
8	NPTN,NRXN1	1	0	Developmental Disorder, Hereditary Disorder, Neurological Disease
9	GLI1,KRT19	1	0	Cancer, Cell Death and Survival, Embryonic Development
10	NANOG,S100A6	1	1	Cell Cycle, Embryonic Development, Cellular Growth and Proliferation
11	A2M,DLG4,GRIN2A,LRP1,MEOX2,PLIN2	1	0	Cancer, Cell Death and Survival, Cell-To-Cell Signaling and Interaction

Table S4. The IPA data in the EMT of KK47 cells.

ID	Molecules in Network	Score	Focus Molecules	Top Diseases and Functions
1	AKT2,ALDH1A3,BCL3,CAMP,CCND2,CD163,COL1A2,EHD1,EHF,F2,FLNA,HMOX1, ITGA1,ITGA2,ITGA5,ITGAV,ITGB1,ITGB5,LAMC2,MAP3K7,MMP1,MMP14,NEDD9, NFE2L2,PRDM1,SCD,SH3KBP1,SQSTM1,TGFB1,TGFBR2,TGM2,TJP2,TNF,VEGFC,ZFP36L1	16	12	Cancer, Cellular Movement, Tumor Morphology
2	ANG,CCL2,CCL5,CXCL8,CXCR4,ERG,FLNB,FOS,GAPDH,HPCAL1,IFIT2,IFNG,IL6,IL1B, ILK,KRT1,KRT10,LIMS1,LMNA,MAP3K8,MAPK1,MMP19,NCOR2,NEDD4L,OASL, PARVA,PPAP2B,RB1,SERPINB2,TERT,TGFB1,TLR3,TNF,TP63,TREM1	14	11	Cell-To-Cell Signaling and Interaction, Tissue Development, Cell Death and Survival
3	SATB1,SUN2	2	1	Cellular Assembly and Organization, Cellular Function and Maintenance, Molecular Transport
4	MMP25,TPM3	2	1	Developmental Disorder, Hereditary Disorder, Skeletal and Muscular Disorders
5	HIF1A,P4HA2	2	1	Cancer, Cardiovascular System Development and Function, Cell Cycle
6	NANOG,S100A6	2	1	Cell Cycle, Embryonic Development, Cellular Growth and Proliferation
7	CDKN2A,GATA4,MYOCD,TBX5,TPM1	1	1	Cellular Development, Tissue Development, Cellular Growth and Proliferation
